# The association between chronic rhinosinusitis and proton pump inhibitor use: a nested case–control study using a health screening cohort

**DOI:** 10.1038/s41598-022-13271-5

**Published:** 2022-06-10

**Authors:** Hyo Geun Choi, Il Gyu Kong

**Affiliations:** 1 Department of Otorhinolaryngology-Head and Neck Surgery, Hallym University College of Medicine, Anyang, 14068 South Korea; 2 Department of Otorhinolaryngology, Hospital Medicine Center, Seoul National University Hospital, 101, Daehak-ro, Jongno-gu, Seoul, 03080 South Korea

**Keywords:** Respiratory tract diseases, Epidemiology, Diseases, Medical research, Signs and symptoms

## Abstract

This study aimed to evaluate the relationship between chronic rhinosinusitis (CRS) and proton pump inhibitor (PPI) use in a Korean population. The Korea National Health Insurance Service-National Sample Cohort was assessed from 2002 to 2013. Patients with CRS (*n* = 7194) and control participants (*n* = 28,776) were matched by random order at a 1:4 ratio for age, sex, income group, region of residence, and index date. We analyzed PPI use by patients with and without CRS. ICD-10 codes defined CRS, and claim codes defined previous PPI use. Conditional logistic regression analyzed the crude and adjusted odds ratios (ORs) with 95% confidence intervals (CI). Subgroup analyses were performed according to age and sex. There was a difference in PPI prescription history and prescription duration between the CRS and control groups. The rate of CRS was higher in current (33.8% [263/778]) and past (26.3% [713/2708]) PPI users than PPI non-users (19.1% [6218/32,484], *P* < 0.001). The adjusted OR (aOR) of CRS with/without nasal polyps was 1.71 (95% CI 1.46–2.02, *P* < 0.001) and 1.28 (95% CI 1.16–1.41, *P* < 0.001) in current and past PPI users, respectively. Irrespective of PPI prescription days, PPI use was associated with higher CRS occurrence (aOR 1.46; 95% CI 1.26–1.69, *P* < 0.001) in the 30–89-day PPI user group. The subgroup analyses results were consistent. The ORs of CRS were higher in PPI users than in the controls, and consistently so in all age and sex groups.

## Introduction

Chronic rhinosinusitis (CRS) and gastroesophageal reflux diseases (GERD) are prevalent disorders that need long-term treatment. The reported prevalence of GERD is as high as 10–20% in Western countries, 2.5–3.8% in Asia, and 4.6–7.3% in Korea^1,2^. The reported prevalence of CRS was 10.9% in the European population, 14% in the USA^3,4^, and, 6–10% in the general population in Korea^5,6^.

The possible link between CRS and GERD has been debated for decades following the increase in their co-occurrence. Epidemiological studies have shown an increased odds ratio (OR) for sinusitis in the presence of reflux esophagitis^7,8^. Whether GERD could be a contributing factor for CRS in some patients has been disputed for decades^9^. However, it was reported that patients with CRS had a significantly higher incidence of GERD than normal controls^10^. A study on a Korean population reported an OR of 2.04 for GERD in patients with CRS^11^. The presence of GERD has been reported as a poor prognostic factor for patients with CRS treated by endoscopic sinus surgery^12^. GERD often manifests with extraesophageal syndromes such as chronic cough, laryngitis, or sinusitis^13^. GERD extraesophageal symptoms include the feeling of mucus in the throat and chronic cough that overlaps with symptoms of CRS.

Although the routine treatment of GERD is not recommended for patients with CRS, acid suppression therapy with proton pump inhibitors (PPIs) is widely applied in patients with CRS because of the common co-occurrence of GERD and CRS. Several studies suggested that PPIs have an anti-inflammatory effect. PPIs were shown to inhibit the interleukin (IL)-4 and IL-13 signaling through the signal transducer and activator of the transcription (STAT) 6 signaling pathway in a murine asthma model. Omeprazole blocked the expression Th2 cytokine-induced eotaxin-3 in vitro esophageal epithelial cell culture experiment^14,15^. IL-13-induced eotaxin-3 in HNECs and BEAS-2B cells was significantly inhibited by PPIs, and the in vivo levels of eotaxin-3 in patients with CRS with nasal polyps (CRSwNP) taking PPIs was lower than without PPIs. Furthermore, the H^+^, K^+^-exchange enhanced by IL-13 was blocked by PPIs^16^.

As CRS is a multifactorial inflammatory disease comorbid with GERD, it is reasonable to investigate the association between PPI therapy and CRS, which was rarely studied. This study aimed to determine whether PPI prescription history and duration of treatment affected the CRS risk in the Korean adult population using a national sample cohort.

## Materials and methods

### Data source and ethical consideration

We used the Korean National Health Insurance Service-Health Screening Cohort data for this study. A comprehensive description of this cohort is provided officially^17^. Briefly, Korean National Health Insurance Service-Health Screening Cohort data includes medical care histories indexed by the codes of diagnosis and treatment and provides socioeconomic data, life/death records from 2002 to 2015. The cohort data were collected until an event of death occurred. The Institutional Review Board (IRB) Ethics Committee of Hallym University approved the study (IRB No: 2019-10-023). All the analyses were conducted with strict adherence to the guidelines and regulations of the Ethics Committee. The IRB Ethics Committee exempted the requirement for written informed consent.

### Participant selection

**Figure 1:**
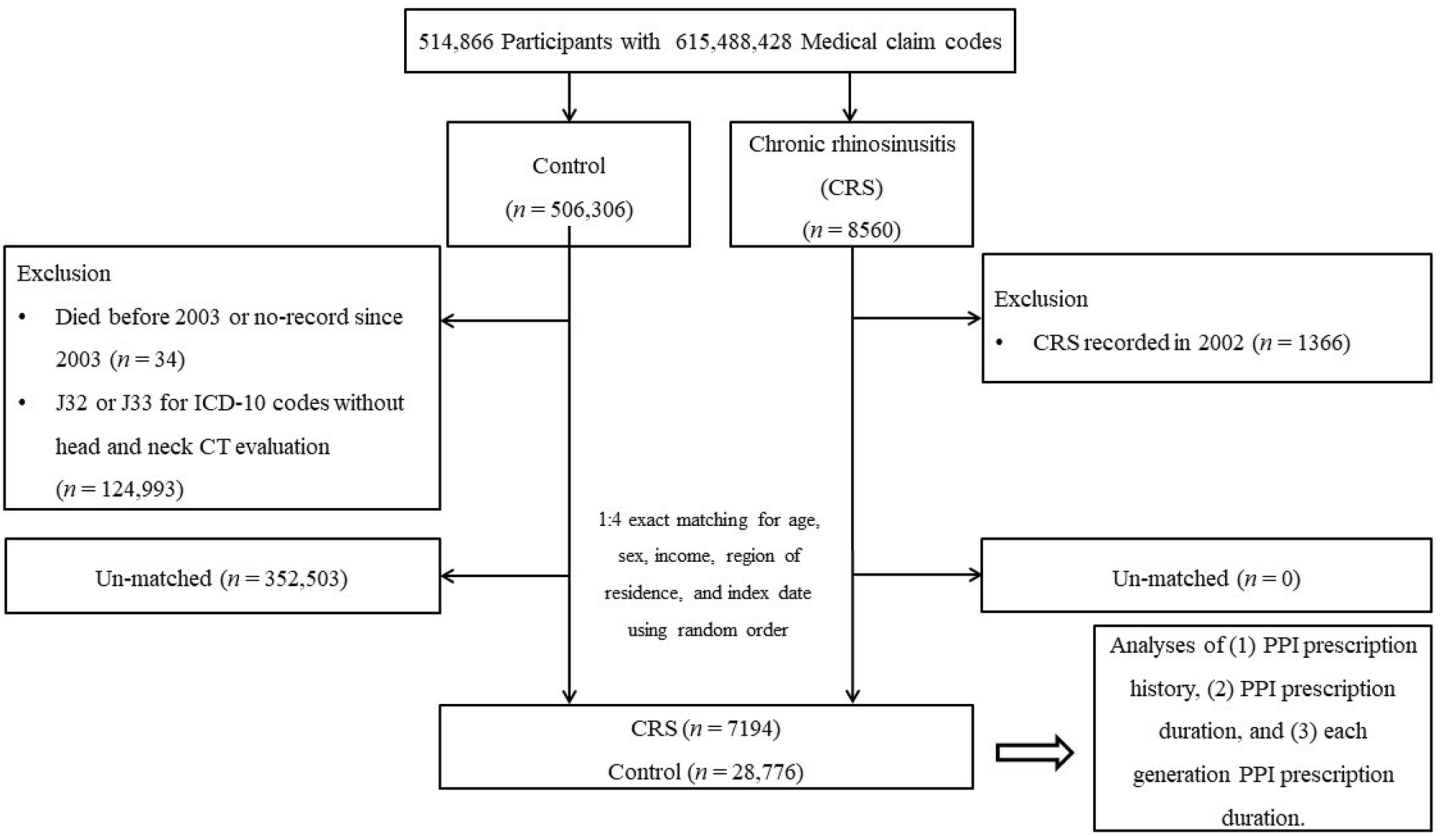
Schematic illustration of the participant selection process that was used in the present study. Of a total of 514,866 participants, 7194 chronic rhinosinusitis (CRS) participants were matched with 28,776 control participants with respect to age, group, sex, income, residence region of, and past medical histories.

Among total 514,866 with 615,488,428 medical claim codes, we selected as the CRS group 8560 patients fulfilling our CRS definition. The remaining 506,306 participants acted as the control group. Participants diagnosed with CRS in 2002 were excluded as we selected only participants diagnosed with CRS for the first time (washout periods, *n* = 1366). We excluded from the control group participants who died before 2003 or no record since 2003 (*n* = 34) or were treated with ICD-10 codes J32 or J33 without head and neck CT evaluations (*n* = 124,993). The CRS and control participants were matched at a ratio 1:4 for age, sex, income, and region of residence. To minimize the selection bias, we used the random number order to select the control participants. The index date of each CRS group participant was set as the time of treatment for CRS. The index date of the control participants was set as the index date of their matched CRS participants. Therefore, each CRS participant and the matched control participants had the same index date. During the matching process, 352,503 of the control participants were excluded. Finally, 7194 participants with CRS were 1:4 matched with 28,776 control participants (Fig. 1).

### Proton pump inhibitor (exposure)

In Korea, PPI is a prescription-only medicine. We defined PPI users as participants prescribed PPI within a year before the index date. PPI users were classified into three categories based on: (1) PPI prescription history, (2) PPI prescription duration, and (3) prescription duration for each PPI generation.

In the first category, the participants were grouped as current PPI users (PPI prescribed PPI within 30 days before the index date), past PPI users (PPI prescribed 31–365 days before the index date) or PPI non-users^18^.

In the second category, the participants were grouped into four groups according to the PPI prescription duration as follows: (1) PPI non-users, (2) PPI prescribed for < 30 days, (3) PPI prescribed for 30–89 days, and (4) PPI prescribed for ≥ 90 days.

In the third category, the participants were categorized into two categories according to the PPI generations. First generation PPIs included lansoprazole, omeprazole, and pantoprazole. Second generation PPIs included dexlansoprazole, esomeprazole, ilaprazole, and rabeprazole. PPI prescription duration were grouped within each PPI generation, considered a continuous variable for analysis.

### Chronic rhinosinusitis (outcome)

CRS was defined based on the ICD-10 codes (J32). Among these, we defined CRS patients who visited clinics at least twice and who underwent head and neck CT evaluation (with Claim codes: HA401–HA416, HA441–HA443, HA451–HA453, HA461–HA463, or HA471–HA473). Among the patients with CRS, 3578 were treated for nasal polyps (J33), and other 3616 participants were treated for CRS without nasal polyps.

### Covariates

The participants were divided into ten age groups of 5-year intervals, starting with the 40–44 years group and ending with an 85+ years groups^19^. The patients were divided into five income classes (Class 1, lowest income to Class 5, highest income). The residence region was classified as urban and rural, as per our previous study^19^. Smoking, alcohol consumption, and obesity based on the body mass index (BMI, kg/m^2^) were categorized in following our study^20^. Data on total cholesterol (mg/dL), systolic blood pressure (SBP, mmHg), diastolic blood pressure (DBP, mmHg), and fasting blood glucose (mg/dL) were retrieved. We used the Charlson Comorbidity Index (CCI), except for respiratory diseases^21^.

Treatment for GERD (ICD-10 codes K21) was assessed during the year (365 days) before the index date.

Asthma and chronic obstructive pulmonary disease (COPD) were defined following our previous studies^22–25^.

We summed the data on the prescription duration on steroids, H2 blockers, and non-steroidal anti-inflammatory drugs (NSAIDs) during the year (365 days) before the index date.

### Statistical analyses

The general characteristics of the CRS and control groups were compared using the chi-square test for categorical variables and Wilcoxon rank-sum test for continuous variables.

We used conditional logistic regression to analyze the odds ratios (ORs) and 95% confidence intervals (CIs) of PPI prescription history, PPI prescription duration per PPI generation for CRS. In these analyses, we calculated the crude model, model 1 (adjusted for total cholesterol, SBP, DBP, and fasting blood glucose), model 2 (adjusted for all variables in model 1 plus obesity, smoking, alcohol consumption, CCI score, asthma, and COPD), and model 3 (adjusted for all variables in model 2 plus the number of GERD treatments, steroid prescription duration, NSAID prescription duration, and H2 blocker prescription duration) were calculated. The analyses were stratified by age, sex, income, and residence region. We also analyzed the ORs for CRS with and without nasal polyps, respectively (Supplementary Tables S1, S2). The ORs for prescription duration of each generation PPI were calculated per 365 days of prescription, where the ORs per 1 day of prescription converged to 1.0.

For the subgroup analyses, we divided participants by age (< 60 years old and ≥ 60 years old), sex (males and females), income (low income and high income), and residence region (urban and rural) using the model 3 with conditional analyses.

Additionally, we performed unconditional logistic regression subgroup analyses according to obesity, smoking, alcohol consumption, total cholesterol, blood pressure, fasting blood glucose, CCI score, the number of GERD treatments, asthma, COPD, steroid prescription duration, NSAID prescription duration, and H2 blocker prescription duration (Supplementary Table S3).

We used SAS, Version 9.4 (SAS Institute Inc., Cary, NC, USA) for the statistical analyses. All analyses were two-tailed, and the significance level was set to *P* < 0.05.

### Ethics approval and consent to participate

Ethical approval for this study was obtained from the ethics committee of the Institutional Review Board of Hallym University (2019-10-023) and written informed consent was waived.

## Results

### General characteristics of the study population

The participants general characteristics (age, sex, income, and region of residence) were identical due to the 1:4 matching (*P* > 0.99, Table 1). The mean follow-time (standard deviation) for CRS patients and controls are 94.8 (42.5) and 93.5 (43.0) months, respectively. The groups differed in the BMI distribution. The CRS group had lower proportions of underweight, normal, and obese II and higher proportions of overweight and obese I patients than the control (*P* < 0.001). Non-smokers, current smokers, and past smokers were more frequent in the CRS group than the control.

**Table 1:** General characteristics of participants.

Characteristics	Total participants		
	CRS total	Control	P-value
**CRS total (*n*, %)**	7194 (20.0)	28,776 (80.0)	< 0.001*
CRS with nasal polyps (*n*, %)	3578 (49.7)	14,312 (49.7)	< 0.001*
CRS without nasal polyps (*n*, %)	3616 (50.3)	14,464 (50.3)	< 0.001*
**Age (years old, *n*, %)**			1.000
40–44	424 (5.9)	1696 (5.9)	
45–49	1224 (17.0)	4896 (17.0)	
50–54	1537 (21.4)	6148 (21.4)	
55–59	1468 (20.4)	5872 (20.4)	
60–64	1108 (15.4)	4432 (15.4)	
65–69	770 (10.7)	3080 (10.7)	
70–74	424 (5.9)	1696 (5.9)	
75–79	178 (2.5)	712 (2.5)	
80–84	50 (0.7)	200 (0.7)	
85+	11 (0.2)	44 (0.2)	
**Sex (*n*, %)**			1.000
Male	4387 (61.0)	17,548 (61.0)	
Female	2807 (39.0)	11,228 (39.0)	
**Income (*n*, %)**			1.000
1 (lowest)	868 (12.1)	3472 (12.1)	
2	847 (11.8)	3388 (11.8)	
3	1096 (15.2)	4384 (15.2)	
4	1553 (21.6)	6212 (21.6)	
5 (highest)	2830 (39.3)	11,320 (39.3)	
**Residence region (*n*, %)**			1.000
Urban	3367 (46.8)	13,468 (46.8)	
Rural	3827 (53.2)	15,308 (53.2)	
**Obesity (*n*, %)**^**‡**^			< 0.001*
Underweight	125 (1.7)	583 (2.0)	
Normal	2332 (32.4)	10,048 (34.9)	
Overweight	2135 (29.7)	7926 (27.5)	
Obese I	2402 (33.4)	9360 (32.5)	
Obese II	200 (2.8)	859 (3.0)	
**Smoking status (*n*, %)**			< 0.001*
Non-smoker	4748 (66.0)	18,744 (65.1)	
Past smoker	1025 (14.3)	3486 (12.1)	
Current smoker	1421 (19.8)	6546 (22.8)	
**Alcohol consumption (*n*, %)**			0.091
< 1 time a week	4797 (66.7)	18,884 (65.6)	
≥ 1 time a week	2397 (33.3)	9892 (34.4)	
Total cholesterol (mg/dL, mean, SD)	197.4 (37.0)	199.2 (37.7)	< 0.001^†^
SBP (mmHg, mean, SD)	78.5 (10.7)	79.1 (11.0)	< 0.001^†^
DBP (mmHg, mean, SD)	125.4 (16.3)	126.7 (16.9)	< 0.001^†^
Fasting blood glucose (mg/dL, mean, SD)	98.9 (30.7)	100.2 (30.7)	0.009^†^
**CCI score (score, *n*, %)**			< 0.001*
0	4961 (69.0)	21,242 (73.8)	
1	1018 (14.2)	3302 (11.5)	
≥ 2	1215 (16.9)	4232 (14.7)	
Asthma (*n*, %)	2176 (30.3)	4773 (16.6)	< 0.001*
COPD (*n*, %)	791 (11.0)	1,552 (5.4)	< 0.001*
No. of GERD treatments (No., mean, SD)	0.8 (2.5)	0.5 (2.0)	< 0.001^†^
Steroid prescription duration (day, mean, SD)	9.2 (32.2)	5.1 (23.7)	< 0.001^†^
NSAID prescription duration (day, mean, SD)	30.4 (56.9)	21.7 (50.6)	< 0.001^†^
H2 blocker prescription duration (day, mean, SD)	27.7 (59.7)	19.0 (52.9)	< 0.001^†^
**PPI prescription history (*n*, %)**			< 0.001*
PPI non-user	6218 (86.4)	26,266 (91.3)	
Past PPI user	713 (9.9)	1995 (6.9)	
Current PPI user	263 (3.7)	515 (1.8)	
**PPI prescription duration (*n*, %)**			< 0.001*
PPI non-user	6218 (86.4)	26,266 (91.3)	
< 30 days	504 (7.0)	1424 (5.0)	
30–89 days	306 (4.3)	735 (2.6)	
≥ 90 days	166 (2.3)	351 (1.2)	
1st generation PPI prescription duration (day, mean, SD)	5.1 (26.7)	2.7 (18.2)	< 0.001^†^
2nd generation PPI prescription duration (day, mean, SD)	2.2 (17.1)	1.4 (14.4)	< 0.001^†^

*CCI* Charlson comorbidity index, *COPD* chronic obstructive pulmonary disease, *CRS* chronic rhinosinusitis, *DBP* diastolic blood pressure, *GERD* gastro-esophageal reflux disease, *NSAID* non-steroidal anti-inflammatory drug, *PPI* proton pump inhibitor, *SBP* systolic blood pressure, *SD* standard deviation.

*Chi-square test. Significance at *P* < 0.05.

^†^Wilcoxon rank-sum test. Significance at *P* < 0.05.

^‡^Obesity (BMI, body mass index, kg/m^2^) was categorized as < 18.5 (underweight), ≥ 18.5 to < 23 (normal), ≥ 23 to < 25 (overweight), ≥ 25 to < 30 (obese I), and ≥ 30 (obese II).

The total cholesterol level, SBP, DBP, and fasting blood glucose in the CRS group were lower than in the control group. Individuals with CCI scores of 1 and ≥ 2 were more prevalent in the CRS group than in the control group. Higher rates of asthma and COPD were observed in the CRS group (*P* < 0.05 for all, Table 1). The number of GERD treatments was higher in the CRS group than in the control group. The prescription duration of steroids, NSAIDs, H2 blockers were higher in CRS group. Regarding the PPI use history, there are fewer PPI non-users, and more past and current PPI users in CRS group. For the PPI prescription duration, there are more CRS patients in all the usage periods (< 30 days, 30–89 days, ≥ 90 days). The PPI prescription days were longer in CRS, regardless of the PPI generation.

### The association between PPI use and CRS

The adjusted ORs (aORs) for CRS according to PPI prescription history and PPI prescription duration are presented in Table 2. The aOR for CRS in current PPI users was 1.71 (95% CI 1.46–2.02, *P* < 0.001), and 1.28 (95% CI 1.16–1.41, *P* < 0.001) in past PPI users. The aOR for CRS were 1.30 (95% CI 1.05–1.61, *P* < 0.05), 1.46 (95% CI 1.26–1.69, *P* < 0.001) and 1.30 (95% CI 1.05–1.61, *P* < 0.05) in groups with PPI prescription ≥ 90, 30–89 and < 30 days, respectively. The aOR for CRS was increased the 1st generation PPI prescription group (aOR 2.45; 95% CI 1.59–3.76; *P* < 0.001), but not in the 2nd generation PPI prescription group (aOR 1.08; 95% CI 0.59–1.99, *P* > 0.05).

**Table 2:** Odd ratios (95% confidence interval) of CRS according to PPI prescription history/PPI prescription duration/each generation PPI prescription duration.

Characteristics	No. of CRS/no. of participants (%)	Odds ratios (95% confidence intervals)							
		Crude^†^	P-value	Model 1^†‡^	P-value	Model 2^†§^	P-value	Model 3^†‖^	P-value
**PPI prescription history**									
Current PPI user	263/778 (33.8)	2.19 (1.88–2.55)	< 0.001*	2.17 (1.86–2.53)	< 0.001*	2.05 (1.76–2.40)	< 0.001*	1.71 (1.46–2.02)	< 0.001*
Past PPI user	713/2708 (26.3)	1.52 (1.39–1.67)	< 0.001*	1.51 (1.38–1.66)	< 0.001*	1.45 (1.32–1.59)	< 0.001*	1.28 (1.16–1.41)	< 0.001*
PPI non-user (reference)	6218/32,484 (19.1)	1		1		1		1	
**PPI prescription duration**									
≥ 90 days	166/517 (32.1)	2.04 (1.69–2.46)	< 0.001*	2.01 (1.66–2.42)	< 0.001*	1.83 (1.51–2.22)	< 0.001*	1.30 (1.05–1.61)	0.016*
30–89 days	306/1041 (29.4)	1.78 (1.55–2.04)	< 0.001*	1.76 (1.54–2.02)	< 0.001*	1.71 (1.49–1.96)	< 0.001*	1.46 (1.26–1.69)	< 0.001*
< 30 days	504/1928 (26.1)	2.04 (1.69–2.46)	< 0.001*	2.01 (1.66–2.42)	< 0.001*	1.83 (1.51–2.22)	< 0.001*	1.30 (1.05–1.61)	0.016*
PPI non-user (reference)	6218/32,484 (19.1)	1		1		1		1	
1st generation PPI prescription duration	N/A	5.73 (3.87–8.47)	< 0.001*	5.61 (3.79–8.30)	< 0.001*	4.93 (3.31–7.35)	< 0.001*	2.45 (1.59–3.76)	< 0.001*
2nd generation PPI prescription duration	N/A	3.10 (1.81–5.32)	< 0.001*	3.03 (1.76–5.20)	< 0.001*	2.55 (1.46–4.45)	0.001*	1.08 (0.59–1.99)	0.797

*CCI* Charlson comorbidity index, *COPD* chronic obstructive pulmonary disease, *CRS* chronic rhinosinusitis, *DBP* diastolic blood pressure, *GERD* gastro-esophageal reflux disease, *NSAID* non-steroidal anti-inflammatory drug, *PPI* proton pump inhibitor, *SBP* systolic blood pressure.

*Conditional logistic regression model, Significance at *P* < 0.05.

^†^Models stratified by age, sex, income, and residence region.

^‡^Model 1 was adjusted for total cholesterol, SBP, DBP, and fasting blood glucose.

^§^Model 2 was adjusted for all variables in model 1 plus obesity, smoking, alcohol consumption, CCI score, asthma, and COPD.

^‖^Model 3 was adjusted for all variables in model 2 plus the number of GERD treatments, steroid/ NSAID/ H2 blocker prescription duration.

Of note, when analyzing the aOR for CRSwNP and CRS without nasal polyps (CRSsNP) separately according to PPI prescription and PPI prescription duration, current and past PPI use history and prescription duration were associated with a significant increase in the aOR only in patients with CRSsNP (Table 3), showing the different effects of PPI use on CRS according to the presence of nasal polyps.

**Table 3:** Odds ratios (95% confidence intervals) of PPI prescription history/PPI prescription duration/each generation PPI prescription duration for CRS with nasal polyps and for CRS without nasal polyps.

Characteristics	No. of CRS/no. of participants (%)	Odds ratios (95% confidence intervals)							
		Crude^†^	P-value	Model 1^†‡^	P-value	Model 2^†§^	P-value	Model 3^†‖^	P-value
**Odds ratios for CRS with nasal polyps**									
PPI prescription history									
Current PPI user	80/294 (27.2)	1.53 (1.18–1.99)	0.001*	1.52 (1.17–1.98)	0.002*	1.45 (1.11–1.90)	0.006*	1.26 (0.96–1.67)	0.099
Past PPI user	253/1120 (22.6)	1.20 (1.03–1.38)	0.017*	1.19 (1.03–1.38)	0.020*	1.13 (0.97–1.31)	0.113	1.02 (0.87–1.20)	0.798
PPI non-user (reference)	3245/16,476 (19.7)	1		1		1		1	
PPI prescription history									
≥ 90 days	54/191 (28.3)	1.62 (1.18–2.23)	0.003*	1.61 (1.17–2.21)	0.004*	1.41 (1.02–1.95)	0.040*	1.05 (0.73–1.51)	0.785
30–89 days	96/426 (22.5)	1.19 (0.95–1.50)	0.136	1.19 (0.94–1.50)	0.147	1.15 (0.91–1.46)	0.235	1.02 (0.80–1.31)	0.854
< 30 days	183/797 (23.0)	1.22 (1.03–1.45)	0.022*	1.22 (1.03–1.44)	0.027*	1.16 (0.98–1.38)	0.089	1.09 (0.91–1.30)	0.341
PPI non-user (reference)	3245/16,476 (19.7)	1		1		1		1	
1st generation PPI prescription duration	3578/17,890 (20.0)	2.42 (1.19–4.92)	0.015*	2.37 (1.16–4.82)	0.017*	1.84 (0.89–3.82)	0.101	0.97 (0.44–2.14)	0.931
2nd generation PPI prescription duration	3578/17,890 (20.0)	2.89 (0.96–8.68)	0.059	2.86 (0.95–8.60)	0.062	2.28 (0.73–7.10)	0.156	1.18 (0.35–3.96)	0.791
**Odds ratios for CRS without nasal polyps**									
PPI prescription history									
Current PPI user	183/484 (37.8)	2.73 (2.26–3.30)	< 0.001*	2.71 (2.24–3.28)	< 0.001*	2.54 (2.09–3.08)	< 0.001*	2.07 (1.68–2.54)	< 0.001*
Past PPI user	460/1588 (29.0)	1.82 (1.62–2.04)	< 0.001*	1.80 (1.60–2.02)	< 0.001*	1.73 (1.53–1.95)	< 0.001*	1.50 (1.33–1.71)	< 0.001*
PPI non-user (reference)	2973/16,008 (18.6)	1		1		1		1	
PPI prescription history									
≥ 90 days	112/326 (34.4)	2.37 (1.88–3.00)	< 0.001*	2.34 (1.85–2.95)	< 0.001*	2.15 (1.69–2.74)	< 0.001*	1.48 (1.13–1.94)	0.004*
30–89 days	210/615 (34.2)	2.31 (1.95–2.75)	< 0.001*	2.29 (1.93–2.72)	< 0.001*	2.21 (1.85–2.64)	< 0.001*	1.85 (1.54–2.22)	< 0.001*
< 30 days	321/1131 (28.4)	1.76 (1.54–2.02)	< 0.001*	1.75 (1.53–2.01)	< 0.001*	1.68 (1.46–1.93)	< 0.001*	1.54 (1.33–1.77)	< 0.001*
PPI non-user (reference)	2973/16,008 (18.6)	1		1		1		1	
1st generation PPI prescription duration	3616/18,080 (20.0)	8.85 (5.43–14.40)	< 0.001*	8.76 (5.38–14.24)	< 0.001*	8.01 (4.89–13.10)	< 0.001*	3.72 (2.20–6.29)	< 0.001*
2nd generation PPI prescription duration	3616/18,080 (20.0)	3.19 (1.72–5.94)	< 0.001*	3.12 (1.67–5.82)	< 0.001*	2.68 (1.41–5.09)	0.003*	1.04 (0.51–2.13)	0.916

*CCI* Charlson comorbidity index, *COPD* chronic obstructive pulmonary disease, *CRS* chronic rhinosinusitis, *DBP* diastolic blood pressure, *GERD* gastro-esophageal reflux disease, *NSAID* non-steroidal anti-inflammatory drug, *PPI* proton pump inhibitor, *SBP* systolic blood pressure.

*Conditional logistic regression model, Significance at *P* < 0.05.

^†^Models stratified by age, sex, income, and residence region.

^‡^Model 1 was adjusted for total cholesterol, SBP, DBP, and fasting blood glucose.

^§^Model 2 was adjusted for all variables in model 1 plus obesity, smoking, alcohol consumption, CCI score, asthma, and COPD.

^‖^Model 3 was adjusted for all variables in model 2 plus the number of GERD treatments, steroid/NSAID/H2 blocker prescription duration.

As presented in Table 3, PPI prescription was associated with the risk of CRSsNP with a significant increase in the aOR in current PPI users (aOR 2.07; 95% CI 1.68–2.54; *P* < 0.001), past PPI use (aOR 1.50; 95% CI 1.33–1.71, *P* < 0.001), PPI prescription ≥ 90 days (aOR 1.48; 95% CI 1.13–1.94, *P* = 0.004, Table 3), 30–89 days (aOR 1.85; 95% CI 1.54–2.22, *P* < 0.001), < 30 days (aOR 1.54; 95% CI 1.33–1.77, *P* < 0.001), and first generation PPI prescription duration (aOR 3.72; 95% CI 2.20–6.29, *P* < 0.001). The aORs of CRSwNP were not associated with PPI prescription history or prescription duration.

The subgroup analysis for each factor showed consistent results, with a common significant association between current PPI use / first generation PPI prescription duration and the aORs > 1 for CRS (Supplementary Tables S1–S5).

## Discussion

We presented a study of the association between PPI use and the subsequent occurrence of CRS. We have demonstrated that the aOR for CRSsNP was higher in current and past PPI users than in the matched control group. These results were consistent in the subgroup analysis after adjusting for age, sex, comorbidities such as GERD, and medications. Irrespective of its duration, PPI use was associated with an increased OR for CRS.

Evaluating whether PPI use affected the CRS risk is important because of the generalized use of PPI in CRS treatment, and long-term PPI treatment has been reported harmful to various health conditions. Long-term PPI use could increase the risk of serious side effects, including serious infections (pneumonia, intestinal infections), micronutrient deficiencies, kidney disease, osteoporosis, acid rebound after discontinuation of treatment, and cardiovascular disease^26–29^. Further to the previously reported adverse effects on health, we demonstrated in this study that PPI use was associated with an increase in the OR for CRS.

This study started from the widespread use of PPIs and their anti-inflammatory effects. Contrary to actual estimates, CRS increased with PPI use, the rationale of which is unclear.

An increase in infections due to the suppression of gastric acid secretion has been investigated in intestinal infectious diseases, reporting an increased risk for infection during long-term PPI use. Studies showed a twofold increase in the risk for spontaneous bacterial peritonitis in association with PPI use^30^. Systematic reviews showed that PPI use was associated with enteral bacterial infection and intestinal bacterial overgrowth^31–33^. In a meta-analysis of enteric infections associated with PPI use in over 10,000 patients, a pooled OR of 3.33 was reported^32^. In a more recent randomized controlled trial, the enteric infection rate was significantly increased in participants treated with pantoprazole (1.4% vs 1.0% in the placebo group; OR 1.33; 95% CI 1.01–1.75)^34^. Adjusted relative risks reported for *Salmonella* in two studies were 4.2–8.3^31^. The adjusted relative risk ranges for *Campylobacter* and *Clostridium difficile* infections with PPI use were 3.5–11.7 (4 studies) and 1.2–5.0 (17/ 27 studies)^31^. Studies reported that enteric viral infection increased in PPI users^35^. Moreover, a recent study found that PPI use was related to COVID-19 positivity in a dose-dependent relationship, with an aOR of 3.67 for PPI use twice daily and 2.15 for use up to once daily^36^. More surprisingly, PPI might affect the oropharyngeal microbiome, which increases the risk for pneumonia^37^. Some observational studies reported the PPIs were associated with increased risk for community-acquired pneumonia^38^. Although investigated in an in vitro study, PPI inhibited the function of neutrophils, which could further increase the risk of bacterial pneumonia^39^.

PPI-associated increased risk of infection could be explained by the possible PPI-induced hypochlorhydria that weakens the host’s natural inhibitory ability against ingested enterobacterial pathogens. This might lead to alteration in the gut microbiota, resulting in intestinal permeability changes, microbial translocation across the intestinal wall, and aspiration of nasopharyngeal or gastric contents with the more pathogenic bacterial burden into the lungs. CRS is a multifactorial disease with a wide spectrum of phenotypes, and there is a clear category, infection-related CRS among them. The etiology of infectious CRS is secondary to chronic bacterial infection, generally followed by viral upper respiratory infection. Some types of CRS are known to occur as a result of infection. The increased risk of infectious diseases associated with PPI use might contribute to the occurrence of CRS in PPI users.

Besides infectious causes of CRS, alteration of the microbial components was widely implicated in CRS pathogenesis, with a potential link between PPI-induced intestinal dysbiosis and subsequent occurrence of CRS, although a direct investigation of the association between dysbiosis and CRS pathogenesis and the effect of PPI on the nasal microbiome is still needed. CRS phenotypes were association with distinct microbial agents^40–42^. Microbiome profiling of patients with CRSsNP by 16SrRNA gene sequencing showed decreased diversity and anaerobic enrichment^41^. Intraoperative sinus brushing from 59 patients with CRS and ten controls revealed that patients with CRS clustered into distinct sub-groups dominated by a pathogenic family such as Streptococcaceae, Pseudomonadaceae, Corynebacteriaceae, or Staphylococcaceae^43^.

This study had several limitations. First, we performed corrections for GERD, NSAIDs prescription, and H2 blocker that may accompany PPI, but considering the wide range of PPI uses and many confounding factors with possible association with CRS, it is possible that factors not included in the analyses (e.g. allergic rhinitis, pylori infections, esophagitis and peptic ulcer diseases) might have affected the occurrence of CRS. It is conceivable that treatment of preceding upper respiratory tract infections and the use of antibiotics may have influenced the prescription of PPIs. Second, the second-generation PPIs did not affect the occurrence of CRS in our cohort, but we do have a suitable explanation for this. The two PPI categories may have different associations with CRS occurrence due to differences in their pharmacological action. However, factors not investigated in this study, such as doctors’ prescription behavior related to the second-generation PPIs as a newer drug than first-generation PPI, might have influenced the results. Third, we did not include data on the use of other acid-suppressive medications such as H2 blockers. It is unknown whether their use was associated with an elevated risk for subsequent CRS. Fourth, we could suggest a protopathic bias of this study in that PPI use may be the marker of undescribed acute events such as hospitalization due to acute illness or prescription for the early preceding symptoms of CRS. Fifth, there is a possibility that patients with CRS who did not seek the medical treatment might be included in control group because we defined CRS with ICD-10 codes that were issued when they visited clinics, considering relatively low mortality rate of CRS, and this could underestimate the actual risk of CRS.

## Conclusion

This study confirmed that patients with current or past PPI use were associated with a higher risk for CRS than was the matched control group, regardless of the prescription duration. Considering diverse indications for PPI prescription, our findings highlight the need for clinicians to be aware of the risk associated with PPI use in patients irrespective of the age or sex of their patients. Further research on the pathophysiological link between PPI use and CRS is required.

## Supplementary Information

Supplementary Tables.
