# Supplementary material for: The association between chronic rhinosinusitis and proton pump inhibitor use: a nested case–control study using a health screening cohort
Source: Sci Rep. 2022 Jun 10;12:9554. doi: 10.1038/s41598-022-13271-5 (PMC9187650; doi:10.1038/s41598-022-13271-5)
Supplement: Supplementary file 1 — Supplementary Tables. [file 41598_2022_13271_MOESM1_ESM.docx]

**S1 Table** Subgroup analyses of PPI prescription history/PPI prescription duration/each generation PPI prescription duration for CRS according to covariates in model 3

| Characteristics | | | | No. of CRS/ No. of participants (%) | aORs (95% CIs) | P-value |
| --- | --- | --- | --- | --- | --- | --- |
| **Age < 60 years old (n = 23,265)** † | | | |  |  |  |
|  |  | PPI prescription history | |  |  |  |
|  |  |  | Current PPI user | 132/368 (35.9) | 1.86 (1.48-2.35) | <0.001* |
|  |  |  | Past PPI user | 368/1,394 (26.4) | 1.23 (1.08-1.41) | 0.003* |
|  |  |  | PPI non-user (reference) | 4,153/21,503 (19.3) | 1 |  |
|  |  | PPI prescription duration | |  |  |  |
|  |  |  | ≥ 90 days | 67/188 (35.6) | 1.35 (0.95-1.90) | 0.092 |
|  |  |  | 30 – 89 days | 145/514 (28.2) | 1.35 (1.10-1.67) | 0.005* |
|  |  |  | < 30 days | 288/1,060 (27.2) | 1.34 (1.16-1.56) | <0.001* |
|  |  |  | PPI non-user (reference) | 4,153/21,503 (19.3) | 1 |  |
|  |  | 1^st^ generation PPI prescription duration | | N/A | 3.05 (1.44-6.44) | 0.004* |
|  |  | 2^nd^ generation PPI prescription duration | | N/A | 1.16 (0.38-3.58) | 0.794 |
| **Age ≥ 60 years old (n = 12,705)** | | | |  |  |  |
|  |  | PPI prescription history | |  |  |  |
|  |  |  | Current PPI user | 131/410 (32.0) | 1.61 (1.28-2.02) | <0.001* |
|  |  |  | Past PPI user | 345/1,314 (26.3) | 1.31 (1.14-1.51) | <0.001* |
|  |  |  | PPI non-user (reference) | 2,065/10,981 (18.8) | 1 |  |
|  |  | PPI prescription duration | |  |  |  |
|  |  |  | ≥ 90 days | 99/329 (30.1) | 1.30 (0.99-1.71) | 0.060 |
|  |  |  | 30 – 89 days | 161/527 (30.6) | 1.57 (1.29-1.93) | <0.001* |
|  |  |  | < 30 days | 216/868 (24.9) | 1.29 (1.09-1.52) | 0.003* |
|  |  |  | PPI non-user (reference) | 2,065/10,981 (18.8) | 1 |  |
|  |  | 1^st^ generation PPI prescription duration | | N/A | 2.32 (1.38-3.90) | 0.002* |
|  |  | 2^nd^ generation PPI prescription duration | | N/A | 1.07 (0.52-2.21) | 0.852 |
| **Males (n = 21,935) †** | | | |  |  |  |
|  |  | PPI prescription history | |  |  |  |
|  |  |  | Current PPI user | 161/468 (34.4) | 1.84 (1.49-2.27) | <0.001* |
|  |  |  | Past PPI user | 436/1,625 (26.8) | 1.36 (1.20-1.54) | <0.001* |
|  |  |  | PPI non-user (reference) | 3,790/19,842 (19.1) | 1 |  |
|  |  | PPI prescription duration | |  |  |  |
|  |  |  | ≥ 90 days | 106/330 (32.1) | 1.44 (1.10-1.89) | 0.009* |
|  |  |  | 30 – 89 days | 181/634 (28.6) | 1.46 (1.21-1.76) | <0.001* |
|  |  |  | < 30 days | 310/1,129 (27.5) | 1.45 (1.26-1.67) | <0.001* |
|  |  |  | PPI non-user (reference) | 3,790/19,842 (19.1) | 1 |  |
|  |  | 1^st^ generation PPI prescription duration | | N/A | 2.55 (1.50-4.33) | 0.001* |
|  |  | 2^nd^ generation PPI prescription duration | | N/A | 1.35 (0.63-2.88) | 0.437 |
| **Females (n = 14,035)** | | | |  |  |  |
|  |  | PPI prescription history | |  |  |  |
|  |  |  | Current PPI user | 102/310 (32.9) | 1.57 (1.21-2.04) | 0.001* |
|  |  |  | Past PPI user | 277/1,083 (25.6) | 1.16 (0.99-1.36) | 0.067 |
|  |  |  | PPI non-user (reference) | 2,428/12,642 (19.2) | 1 |  |
|  |  | PPI prescription duration | |  |  |  |
|  |  |  | ≥ 90 days | 60/187 (32.1) | 1.17 (0.83-1.66) | 0.377 |
|  |  |  | 30 – 89 days | 125/407 (30.7) | 1.45 (1.15-1.83) | 0.002* |
|  |  |  | < 30 days | 194/799 (24.3) | 1.17 (0.98-1.39) | 0.087 |
|  |  |  | PPI non-user (reference) | 2,428/12,642 (19.2) | 1 |  |
|  |  | 1^st^ generation PPI prescription duration | | N/A | 2.45 (1.18-5.06) | 0.016* |
|  |  | 2^nd^ generation PPI prescription duration | | N/A | 0.74 (0.26-2.08) | 0.563 |
| **Low income (n = 14,055) †** | | | |  |  |  |
|  |  | PPI prescription history | |  |  |  |
|  |  |  | Current PPI user | 120/333 (36.0) | 1.84 (1.44-2.35) | <0.001* |
|  |  |  | Past PPI user | 289/1,091 (26.5) | 1.26 (1.08-1.47) | 0.004* |
|  |  |  | PPI non-user (reference) | 2,402/12,631 (19.0) | 1 |  |
|  |  | PPI prescription duration | |  |  |  |
|  |  |  | ≥ 90 days | 74/241 (30.7) | 1.26 (0.92-1.72) | 0.149 |
|  |  |  | 30 – 89 days | 137/432 (31.7) | 1.59 (1.27-1.98) | <0.001* |
|  |  |  | < 30 days | 198/751 (26.4) | 1.29 (1.09-1.54) | 0.004* |
|  |  |  | PPI non-user (reference) | 2,402/12,631 (19.0) | 1 |  |
|  |  | 1^st^ generation PPI prescription duration | | N/A | 2.32 (1.26-4.29) | 0.007* |
|  |  | 2^nd^ generation PPI prescription duration | | N/A | 0.66 (0.27-1.63) | 0.372 |
| **High income (n = 21,915) †** | | | |  |  |  |
|  |  | PPI prescription history | |  |  |  |
|  |  |  | Current PPI user | 143/445 (32.1) | 1.62 (1.30-2.01) | <0.001* |
|  |  |  | Past PPI user | 424/1,617 (26.2) | 1.29 (1.14-1.46) | <0.001* |
|  |  |  | PPI non-user (reference) | 3,816/19,853 (19.2) | 1 |  |
|  |  | PPI prescription duration | |  |  |  |
|  |  |  | ≥ 90 days | 92/276 (33.3) | 1.33 (0.99-1.78) | 0.063 |
|  |  |  | 30 – 89 days | 169/609 (27.8) | 1.37 (1.13-1.67) | 0.001* |
|  |  |  | < 30 days | 306/1,177 (26.0) | 1.35 (1.17-1.55) | <0.001* |
|  |  |  | PPI non-user (reference) | 3,816/19,853 (19.2) | 1 |  |
|  |  | 1^st^ generation PPI prescription duration | | N/A | 2.55 (1.40-4.64) | 0.002* |
|  |  | 2^nd^ generation PPI prescription duration | | N/A | 1.72 (0.73-4.07) | 0.215 |
| **Urban (n = 16,835) †** | | | |  |  |  |
|  |  | PPI prescription history | |  |  |  |
|  |  |  | Current PPI user | 104/308 (33.8) | 1.66 (1.28-2.15) | <0.001* |
|  |  |  | Past PPI user | 316/1,190 (26.6) | 1.22 (1.06-1.42) | 0.007* |
|  |  |  | PPI non-user (reference) | 2,947/15,337 (19.2) | 1 |  |
|  |  | PPI prescription duration | |  |  |  |
|  |  |  | ≥ 90 days | 74/212 (34.9) | 1.32 (0.95-1.84) | 0.096 |
|  |  |  | 30 – 89 days | 133/438 (30.4) | 1.47 (1.18-1.84) | 0.001* |
|  |  |  | < 30 days | 213/848 (25.1) | 1.22 (1.03-1.44) | 0.019* |
|  |  |  | PPI non-user (reference) | 2,947/15,337 (19.2) | 1 |  |
|  |  | 1^st^ generation PPI prescription duration | | N/A | 2.80 (1.47-5.32) | 0.002* |
|  |  | 2^nd^ generation PPI prescription duration | | N/A | 0.87 (0.32-2.37) | 0.788 |
| **Rural (n = 19,135) †** | | | |  |  |  |
|  |  | PPI prescription history | |  |  |  |
|  |  |  | Current PPI user | 159/470 (33.8) | 1.76 (1.42-2.17) | <0.001* |
|  |  |  | Past PPI user | 397/1,518 (26.2) | 1.31 (1.15-1.49) | <0.001* |
|  |  |  | PPI non-user (reference) | 3,271/17,147 (19.1) | 1 |  |
|  |  | PPI prescription duration | |  |  |  |
|  |  |  | ≥ 90 days | 92/305 (30.2) | 1.29 (0.97-1.71) | 0.080 |
|  |  |  | 30 – 89 days | 173/603 (28.7) | 1.45 (1.20-1.76) | <0.001* |
|  |  |  | < 30 days | 291/1,080 (26.9) | 1.40 (1.21-1.62) | <0.001* |
|  |  |  | PPI non-user (reference) | 3,271/17,147 (19.1) | 1 |  |
|  |  | 1^st^ generation PPI prescription duration | | N/A | 2.13 (1.19-3.80) | 0.011* |
|  |  | 2^nd^ generation PPI prescription duration | | N/A | 1.26 (0.58-2.71) | 0.562 |

Abbreviations: CCI, Charlson comorbidity index; CIs, confidence intervals; COPD, chronic obstructive pulmonary disease; CRS, chronic rhinosinusitis; DBP, diastolic blood pressure; GERD, gastro-esophageal reflux disease; NSAID, non-steroidal anti-inflammatory drug; ORs, odds ratios; PPI, proton pump inhibitor; SBP, systolic blood pressure

* Significance at *P* < 0.05

† Conditional logistic regression model was used. The model was stratified by age, sex, income, and residence region. The model was adjusted for total cholesterol, SBP, DBP, fasting blood glucose, obesity, smoking, alcohol consumption, CCI score, asthma, COPD, the number of GERD treatments, steroid/ NSAID/ H2 blocker prescription duration (model 3).

‡ Unconditional logistic regression model was used. The model was adjusted for age, sex, income, residence region, total cholesterol, SBP, DBP, fasting blood glucose, obesity, smoking, alcohol consumption, CCI score, asthma, COPD, the number of GERD treatments, steroid/ NSAID/ H2 blocker prescription duration.

**S2 Table** Subgroup analyses of PPI prescription history/PPI prescription duration/each generation PPI prescription duration for CRS according to covariates in model 3

| Characteristics | | | | No. of CRS/ No. of participants (%) | aORs (95% CIs) | P-value |
| --- | --- | --- | --- | --- | --- | --- |
| **Underweight (n = 708) ‡** | | | |  |  |  |
|  |  | PPI prescription history | |  |  |  |
|  |  |  | Current PPI user | 9/22 (40.9) | 5.90 (2.14-16.25) | 0.001* |
|  |  |  | Past PPI user | 13/58 (22.4) | 1.71 (0.84-3.47) | 0.141 |
|  |  |  | PPI non-user (reference) | 103/628 (16.4) | 1 |  |
|  |  | PPI prescription duration | |  |  |  |
|  |  |  | ≥ 90 days | 5/17 (29.4) | 2.50 (0.69-9.02) | 0.162 |
|  |  |  | 30 – 89 days | 8/23 (34.8) | 3.58 (1.31-9.78) | 0.013* |
|  |  |  | < 30 days | 9/40 (22.5) | 1.87 (0.83-4.24) | 0.132 |
|  |  |  | PPI non-user (reference) | 103/628 (16.4) | 1 |  |
|  |  | 1^st^ generation PPI prescription duration | | N/A | 5.36 (0.29-99.90) | 0.261 |
|  |  | 2^nd^ generation PPI prescription duration | | N/A | 40.02 (0.44->999.99) | 0.110 |
| **Normal weight (n = 12,380) ‡** | | | |  |  |  |
|  |  | PPI prescription history | |  |  |  |
|  |  |  | Current PPI user | 93/266 (35.0) | 1.97 (1.49-2.61) | <0.001* |
|  |  |  | Past PPI user | 243/920 (26.4) | 1.34 (1.13-1.59) | 0.001* |
|  |  |  | PPI non-user (reference) | 1,996/11,194 (17.8) | 1 |  |
|  |  | PPI prescription duration | |  |  |  |
|  |  |  | ≥ 90 days | 58/182 (31.9) | 1.29 (0.90-1.87) | 0.168 |
|  |  |  | 30 – 89 days | 111/351 (31.6) | 1.70 (1.32-2.18) | <0.001* |
|  |  |  | < 30 days | 167/653 (25.6) | 1.39 (1.15-1.68) | 0.001* |
|  |  |  | PPI non-user (reference) | 1,996/11,194 (17.8) | 1 |  |
|  |  | 1^st^ generation PPI prescription duration | | N/A | 2.92 (1.40-6.07) | 0.004* |
|  |  | 2^nd^ generation PPI prescription duration | | N/A | 1.26 (0.40-3.93) | 0.697 |
| **Overweight (n = 10,061) ‡** | | | |  |  |  |
|  |  | PPI prescription history | |  |  |  |
|  |  |  | Current PPI user | 73/222 (32.9) | 1.64 (1.21-2.22) | 0.002* |
|  |  |  | Past PPI user | 218/764 (28.5) | 1.40 (1.17-1.67) | <0.001* |
|  |  |  | PPI non-user (reference) | 1,844/9,075 (20.3) | 1 |  |
|  |  | PPI prescription duration | |  |  |  |
|  |  |  | ≥ 90 days | 42/137 (30.7) | 1.27 (0.84-1.91) | 0.258 |
|  |  |  | 30 – 89 days | 93/289 (32.2) | 1.61 (1.23-2.11) | 0.001* |
|  |  |  | < 30 days | 156/560 (27.9) | 1.41 (1.15-1.72) | 0.001* |
|  |  |  | PPI non-user (reference) | 1,844/9,075 (20.3) | 1 |  |
|  |  | 1^st^ generation PPI prescription duration | | N/A | 1.88 (0.80-4.44) | 0.149 |
|  |  | 2^nd^ generation PPI prescription duration | | N/A | 1.23 (0.39-3.84) | 0.727 |
| **Obese (n = 12,821) ‡** | | | |  |  |  |
|  |  | PPI prescription history | |  |  |  |
|  |  |  | Current PPI user | 88/268 (32.8) | 1.43 (1.08-1.90) | 0.014* |
|  |  |  | Past PPI user | 239/966 (24.7) | 1.11 (0.94-1.31) | 0.208 |
|  |  |  | PPI non-user (reference) | 2,275/11,587 (19.6) | 1 |  |
|  |  | PPI prescription duration | |  |  |  |
|  |  |  | ≥ 90 days | 61/181 (33.7) | 1.29 (0.90-1.85) | 0.175 |
|  |  |  | 30 – 89 days | 94/378 (24.9) | 1.10 (0.86-1.42) | 0.445 |
|  |  |  | < 30 days | 172/675 (25.5) | 1.19 (0.99-1.43) | 0.070 |
|  |  |  | PPI non-user (reference) | 2,275/11,587 (19.6) | 1 |  |
|  |  | 1^st^ generation PPI prescription duration | | N/A | 2.38 (1.19-4.74) | 0.014* |
|  |  | 2^nd^ generation PPI prescription duration | | N/A | 0.68 (0.25-1.85) | 0.446 |
| **Non-smoker (n = 23,492) ‡** | | | |  |  |  |
|  |  | PPI prescription history | |  |  |  |
|  |  |  | Current PPI user | 154/491 (31.4) | 1.49 (1.21-1.84) | <0.001* |
|  |  |  | Past PPI user | 457/1,759 (26.0) | 1.22 (1.08-1.38) | 0.001* |
|  |  |  | PPI non-user (reference) | 4,137/21,242 (19.5) | 1 |  |
|  |  | PPI prescription duration | |  |  |  |
|  |  |  | ≥ 90 days | 96/321 (29.9) | 1.14 (0.87-1.50) | 0.346 |
|  |  |  | 30 – 89 days | 192/655 (29.3) | 1.40 (1.16-1.68) | <0.001* |
|  |  |  | < 30 days | 323/1,274 (25.4) | 1.25 (1.09-1.43) | 0.002* |
|  |  |  | PPI non-user (reference) | 4,137/21,242 (19.5) | 1 |  |
|  |  | 1^st^ generation PPI prescription duration | | N/A | 2.13 (1.22-3.72) | 0.008* |
|  |  | 2^nd^ generation PPI prescription duration | | N/A | 0.75 (0.34-1.65) | 0.471 |
| **Past and current smoker (n = 12,478) ‡** | | | |  |  |  |
|  |  | PPI prescription history | |  |  |  |
|  |  |  | Current PPI user | 109/287 (38.0) | 2.18 (1.67-2.84) | <0.001* |
|  |  |  | Past PPI user | 256/949 (27.0) | 1.41 (1.20-1.66) | <0.001* |
|  |  |  | PPI non-user (reference) | 2,081/11,242 (18.5) | 1 |  |
|  |  | PPI prescription duration | |  |  |  |
|  |  |  | ≥ 90 days | 70/196 (35.7) | 1.68 (1.19-2.38) | 0.004* |
|  |  |  | 30 – 89 days | 114/386 (29.5) | 1.63 (1.28-2.07) | <0.001* |
|  |  |  | < 30 days | 181/654 (27.7) | 1.50 (1.24-1.80) | <0.001* |
|  |  |  | PPI non-user (reference) | 2,081/11,242 (18.5) | 1 |  |
|  |  | 1^st^ generation PPI prescription duration | | N/A | 2.94 (1.50-5.78) | 0.002* |
|  |  | 2^nd^ generation PPI prescription duration | | N/A | 2.26 (0.84-6.05) | 0.105 |
| **Alcohol < 1 time a week (n = 23,681) ‡** | | | |  |  |  |
|  |  | PPI prescription history | |  |  |  |
|  |  |  | Current PPI user | 165/498 (33.1) | 1.61 (1.31-1.97) | <0.001* |
|  |  |  | Past PPI user | 434/1,661 (26.1) | 1.22 (1.07-1.38) | 0.002* |
|  |  |  | PPI non-user (reference) | 4,198/21,522 (19.5) | 1 |  |
|  |  | PPI prescription duration | |  |  |  |
|  |  |  | ≥ 90 days | 101/318 (31.8) | 1.23 (0.94-1.62) | 0.130 |
|  |  |  | 30 – 89 days | 185/628 (29.5) | 1.39 (1.15-1.67) | 0.001* |
|  |  |  | < 30 days | 313/1,213 (25.8) | 1.26 (1.10-1.45) | 0.001* |
|  |  |  | PPI non-user (reference) | 4,198/21,522 (19.5) | 1 |  |
|  |  | 1^st^ generation PPI prescription duration | | N/A | 2.51 (1.46-4.32) | 0.001* |
|  |  | 2^nd^ generation PPI prescription duration | | N/A | 0.74 (0.32-1.75) | 0.495 |
| **Alcohol ≥ 1 time a week (n = 12,289) ‡** | | | |  |  |  |
|  |  | PPI prescription history | |  |  |  |
|  |  |  | Current PPI user | 98/280 (35.0) | 1.92 (1.46-2.52) | <0.001* |
|  |  |  | Past PPI user | 279/1,047 (26.7) | 1.39 (1.18-1.63) | <0.001* |
|  |  |  | PPI non-user (reference) | 2,020/10,962 (18.4) | 1 |  |
|  |  | PPI prescription duration | |  |  |  |
|  |  |  | ≥ 90 days | 65/199 (32.7) | 1.44 (1.01-2.04) | 0.044* |
|  |  |  | 30 – 89 days | 121/413 (29.3) | 1.61 (1.27-2.03) | <0.001* |
|  |  |  | < 30 days | 191/715 (26.7) | 1.43 (1.19-1.71) | <0.001* |
|  |  |  | PPI non-user (reference) | 2,020/10,962 (18.4) | 1 |  |
|  |  | 1^st^ generation PPI prescription duration | | N/A | 2.32 (1.15-4.69) | 0.019* |
|  |  | 2^nd^ generation PPI prescription duration | | N/A | 1.62 (0.68-3.84) | 0.279 |

Abbreviations: CCI, Charlson comorbidity index; CIs, confidence intervals; COPD, chronic obstructive pulmonary disease; CRS, chronic rhinosinusitis; DBP, diastolic blood pressure; GERD, gastro-esophageal reflux disease; NSAID, non-steroidal anti-inflammatory drug; ORs, odds ratios; PPI, proton pump inhibitor; SBP, systolic blood pressure

* Significance at *P* < 0.05

† Conditional logistic regression model was used. The model was stratified by age, sex, income, and residence region. The model was adjusted for total cholesterol, SBP, DBP, fasting blood glucose, obesity, smoking, alcohol consumption, CCI score, asthma, COPD, the number of GERD treatments, steroid/ NSAID/ H2 blocker prescription duration (model 3).

‡ Unconditional logistic regression model was used. The model was adjusted for age, sex, income, residence region, total cholesterol, SBP, DBP, fasting blood glucose, obesity, smoking, alcohol consumption, CCI score, asthma, COPD, the number of GERD treatments, steroid/ NSAID/ H2 blocker prescription duration.

**S3 Table** Subgroup analyses of PPI prescription history/PPI prescription duration/each generation PPI prescription duration for CRS according to covariates in model 3

| Characteristics | | | | No. of CRS/ No. of participants (%) | aORs (95% CIs) | P-value |
| --- | --- | --- | --- | --- | --- | --- |
| **Total cholesterol < 200 mg/dL (n = 19,171) ‡** | | | |  |  |  |
|  |  | PPI prescription history | |  |  |  |
|  |  |  | Current PPI user | 153/437 (35.0) | 1.81 (1.47-2.25) | <0.001* |
|  |  |  | Past PPI user | 431/1,489 (29.0) | 1.41 (1.24-1.60) | <0.001* |
|  |  |  | PPI non-user (reference) | 3,393/17,245 (19.7) | 1 |  |
|  |  | PPI prescription duration | |  |  |  |
|  |  |  | ≥ 90 days | 102/307 (33.2) | 1.45 (1.10-1.90) | 0.520 |
|  |  |  | 30 – 89 days | 184/573 (32.1) | 1.62 (1.34-1.96) | 0.021* |
|  |  |  | < 30 days | 298/1,046 (28.5) | 1.44 (1.24-1.66) | 0.390 |
|  |  |  | PPI non-user (reference) | 3,393/17,245 (19.7) | 1 |  |
|  |  | 1^st^ generation PPI prescription duration | | N/A | 2.75 (1.60-4.72) | <0.001* |
|  |  | 2^nd^ generation PPI prescription duration | | N/A | 1.36 (0.62-2.97) | 0.442 |
| **Total cholesterol ≥ 200 to < 240 mg/dL (n = 12,007) ‡** | | | | | | |
|  |  | PPI prescription history | |  |  |  |
|  |  |  | Current PPI user | 84/241 (34.9) | 1.82 (1.35-2.44) | <0.001* |
|  |  |  | Past PPI user | 217/871 (24.9) | 1.24 (1.04-1.48) | 0.015* |
|  |  |  | PPI non-user (reference) | 2,024/10,895 (18.6) | 1 |  |
|  |  | PPI prescription duration | |  |  |  |
|  |  |  | ≥ 90 days | 48/146 (32.9) | 1.29 (0.86-1.94) | 0.789 |
|  |  |  | 30 – 89 days | 89/323 (27.6) | 1.37 (1.05-1.79) | 0.383 |
|  |  |  | < 30 days | 164/643 (25.5) | 1.35 (1.11-1.63) | 0.381 |
|  |  |  | PPI non-user (reference) | 2,024/10,895 (18.6) | 1 |  |
|  |  | 1^st^ generation PPI prescription duration | | N/A | 3.14 (1.35-7.32) | 0.008* |
|  |  | 2^nd^ generation PPI prescription duration | | N/A | 1.01 (0.30-3.42) | 0.987 |
| **Total cholesterol ≥ 240 mg/dL (n = 4,792) ‡** | | | |  |  |  |
|  |  | PPI prescription history | |  |  |  |
|  |  |  | Current PPI user | 26/100 (26.0) | 1.14 (0.68-1.89) | 0.623 |
|  |  |  | Past PPI user | 65/348 (18.7) | 0.87 (0.64-1.18) | 0.367 |
|  |  |  | PPI non-user (reference) | 801/4,344 (18.4) | 1 |  |
|  |  | PPI prescription duration | |  |  |  |
|  |  |  | ≥ 90 days | 16/64 (25.0) | 0.82 (0.41-1.60) | 0.370 |
|  |  |  | 30 – 89 days | 33/145 (22.8) | 1.10 (0.72-1.68) | 0.575 |
|  |  |  | < 30 days | 42/239 (17.6) | 0.85 (0.60-1.21) | 0.206 |
|  |  |  | PPI non-user (reference) | 801/4,344 (18.4) | 1 |  |
|  |  | 1^st^ generation PPI prescription duration | | N/A | 0.80 (0.21-3.05) | 0.739 |
|  |  | 2^nd^ generation PPI prescription duration | | N/A | 0.53 (0.10-2.77) | 0.450 |
| **SBP < 140 mmHg and DBP < 90 mmHg (n = 26,055) ‡** | | | | |  |  |
|  |  | PPI prescription history | |  |  |  |
|  |  |  | Current PPI user | 209/594 (35.2) | 1.77 (1.47-2.13) | <0.001* |
|  |  |  | Past PPI user | 560/2,089 (26.8) | 1.27 (1.14-1.42) | <0.001* |
|  |  |  | PPI non-user (reference) | 4,611/23,372 (19.7) | 1 |  |
|  |  | PPI prescription duration | |  |  |  |
|  |  |  | ≥ 90 days | 126/387 (32.6) | 1.26 (0.99-1.62) | 0.063 |
|  |  |  | 30 – 89 days | 248/811 (30.6) | 1.50 (1.27-1.76) | <0.001* |
|  |  |  | < 30 days | 395/1,485 (26.6) | 1.33 (1.17-1.50) | <0.001* |
|  |  |  | PPI non-user (reference) | 4,611/23,372 (19.7) | 1 |  |
|  |  | 1^st^ generation PPI prescription duration | | N/A | 2.10 (1.25-3.53) | 0.005* |
|  |  | 2^nd^ generation PPI prescription duration | | N/A | 1.18 (0.60-2.34) | 0.637 |
| **SBP ≥ 140 mmHg or DBP ≥ 90 mmHg (n = 9,915) ‡** | | | | |  |  |
|  |  | PPI prescription history | |  |  |  |
|  |  |  | Current PPI user | 54/184 (29.4) | 1.50 (1.06-2.13) | 0.023* |
|  |  |  | Past PPI user | 153/619 (24.7) | 1.28 (1.05-1.57) | 0.017* |
|  |  |  | PPI non-user (reference) | 1,607/9,112 (17.6) | 1 |  |
|  |  | PPI prescription duration | |  |  |  |
|  |  |  | ≥ 90 days | 40/130 (30.8) | 1.46 (0.95-2.24) | 0.084 |
|  |  |  | 30 – 89 days | 58/230 (25.2) | 1.33 (0.96-1.83) | 0.027* |
|  |  |  | < 30 days | 109/443 (24.6) | 1.30 (1.03-1.64) | 0.038* |
|  |  |  | PPI non-user (reference) | 1,607/9,112 (17.6) | 1 |  |
|  |  | 1^st^ generation PPI prescription duration | | N/A | 3.44 (1.62-7.30) | 0.001* |
|  |  | 2^nd^ generation PPI prescription duration | | N/A | 0.79 (0.21-3.02) | 0.730 |
| **Fasting blood glucose < 100 mg/dL (n = 23,085) ‡** | | | | |  |  |
|  |  | PPI prescription history | |  |  |  |
|  |  |  | Current PPI user | 154/471 (32.7) | 1.60 (1.30-1.98) | <0.001* |
|  |  |  | Past PPI user | 444/1,650 (26.9) | 1.29 (1.14-1.46) | <0.001* |
|  |  |  | PPI non-user (reference) | 4,091/20,964 (19.5) | 1 |  |
|  |  | PPI prescription duration | |  |  |  |
|  |  |  | ≥ 90 days | 94/298 (31.5) | 1.24 (0.93-1.65) | 0.138 |
|  |  |  | 30 – 89 days | 190/636 (29.9) | 1.47 (1.22-1.76) | <0.001* |
|  |  |  | < 30 days | 314/1,187 (26.5) | 1.32 (1.15-1.51) | <0.001* |
|  |  |  | PPI non-user (reference) | 4,091/20,964 (19.5) | 1 |  |
|  |  | 1^st^ generation PPI prescription duration | | N/A | 2.26 (1.29-3.97) | 0.005* |
|  |  | 2^nd^ generation PPI prescription duration | | N/A | 0.79 (0.33-1.88) | 0.600 |
| **Fasting blood glucose ≥ 100 mg/dL (n = 12,885) ‡** | | | | |  |  |
|  |  | PPI prescription history | |  |  |  |
|  |  |  | Current PPI user | 109/307 (35.5) | 1.90 (1.47-2.47) | <0.001* |
|  |  |  | Past PPI user | 269/1,058 (25.4) | 1.26 (1.07-1.47) | 0.005* |
|  |  |  | PPI non-user (reference) | 2,127/11,520 (18.5) | 1 |  |
|  |  | PPI prescription duration | |  |  |  |
|  |  |  | ≥ 90 days | 72/219 (32.9) | 1.40 (1.01-1.95) | 0.042* |
|  |  |  | 30 – 89 days | 116/405 (28.6) | 1.45 (1.15-1.84) | 0.002* |
|  |  |  | < 30 days | 190/741 (25.6) | 1.34 (1.12-1.60) | 0.002* |
|  |  |  | PPI non-user (reference) | 2,127/11,520 (18.5) | 1 |  |
|  |  | 1^st^ generation PPI prescription duration | | N/A | 2.83 (1.46-5.47) | 0.002* |
|  |  | 2^nd^ generation PPI prescription duration | | N/A | 1.48 (0.63-3.48) | 0.364 |

Abbreviations: CCI, Charlson comorbidity index; CIs, confidence intervals; COPD, chronic obstructive pulmonary disease; CRS, chronic rhinosinusitis; DBP, diastolic blood pressure; GERD, gastro-esophageal reflux disease; NSAID, non-steroidal anti-inflammatory drug; ORs, odds ratios; PPI, proton pump inhibitor; SBP, systolic blood pressure

* Significance at *P* < 0.05

† Conditional logistic regression model was used. The model was stratified by age, sex, income, and residence region. The model was adjusted for total cholesterol, SBP, DBP, fasting blood glucose, obesity, smoking, alcohol consumption, CCI score, asthma, COPD, the number of GERD treatments, steroid/ NSAID/ H2 blocker prescription duration (model 3).

‡ Unconditional logistic regression model was used. The model was adjusted for age, sex, income, residence region, total cholesterol, SBP, DBP, fasting blood glucose, obesity, smoking, alcohol consumption, CCI score, asthma, COPD, the number of GERD treatments, steroid/ NSAID/ H2 blocker prescription duration.

**S4 Table** Subgroup analyses of PPI prescription history/PPI prescription duration/each generation PPI prescription duration for CRS according to covariates in model 3

| Characteristics | | | | No. of CRS/ No. of participants (%) | aORs (95% CIs) | P-value |
| --- | --- | --- | --- | --- | --- | --- |
| **CCI score = 0 (n = 26,203) ‡** | | | |  |  |  |
|  |  | PPI prescription history | |  |  |  |
|  |  |  | Current PPI user | 155/467 (33.2) | 1.82 (1.47-2.24) | <0.001* |
|  |  |  | Past PPI user | 436/1,763 (24.7) | 1.26 (1.12-1.43) | <0.001* |
|  |  |  | PPI non-user (reference) | 4,370/23,973 (18.2) | 1 |  |
|  |  | PPI prescription duration | |  |  |  |
|  |  |  | ≥ 90 days | 88/283 (31.1) | 1.32 (0.99-1.77) | 0.061 |
|  |  |  | 30 – 89 days | 181/658 (27.5) | 1.46 (1.21-1.75) | <0.001* |
|  |  |  | < 30 days | 322/1,289 (25.0) | 1.33 (1.16-1.52) | <0.001* |
|  |  |  | PPI non-user (reference) | 4,370/23,973 (18.2) | 1 |  |
|  |  | 1^st^ generation PPI prescription duration | | N/A | 2.79 (1.49-5.22) | 0.001* |
|  |  | 2^nd^ generation PPI prescription duration | | N/A | 1.15 (0.50-2.63) | 0.747 |
| **CCI score = 1 (n = 4,320) ‡** | | | |  |  |  |
|  |  | PPI prescription history | |  |  |  |
|  |  |  | Current PPI user | 49/119 (41.2) | 1.74 (1.15-2.63) | 0.009* |
|  |  |  | Past PPI user | 122/410 (29.8) | 1.18 (0.92-1.52) | 0.185 |
|  |  |  | PPI non-user (reference) | 847/3,791 (22.3) | 1 |  |
|  |  | PPI prescription duration | |  |  |  |
|  |  |  | ≥ 90 days | 31/89 (34.8) | 1.03 (0.62-1.74) | 0.902 |
|  |  |  | 30 – 89 days | 56/159 (35.2) | 1.45 (1.01-2.09) | 0.044* |
|  |  |  | < 30 days | 84/281 (29.9) | 1.26 (0.95-1.67) | 0.106 |
|  |  |  | PPI non-user (reference) | 847/3,791 (22.3) | 1 |  |
|  |  | 1^st^ generation PPI prescription duration | | N/A | 2.66 (1.00-7.10) | 0.051 |
|  |  | 2^nd^ generation PPI prescription duration | | N/A | 0.48 (0.10-2.31) | 0.358 |
| **CCI score ≥ 2 (n = 5,447) ‡** | | | |  |  |  |
|  |  | PPI prescription history | |  |  |  |
|  |  |  | Current PPI user | 59/192 (30.7) | 1.47 (1.04-2.06) | 0.027* |
|  |  |  | Past PPI user | 155/535 (29.0) | 1.33 (1.07-1.65) | 0.010* |
|  |  |  | PPI non-user (reference) | 1,001/4,720 (21.2) | 1 |  |
|  |  | PPI prescription duration | |  |  |  |
|  |  |  | ≥ 90 days | 47/145 (32.4) | 1.48 (0.99-2.20) | 0.055 |
|  |  |  | 30 – 89 days | 69/224 (30.8) | 1.41 (1.03-1.93) | 0.030* |
|  |  |  | < 30 days | 98/358 (27.4) | 1.30 (1.01-1.67) | 0.044* |
|  |  |  | PPI non-user (reference) | 1,001/4,720 (21.2) | 1 |  |
|  |  | 1^st^ generation PPI prescription duration | | N/A | 1.88 (0.90-3.95) | 0.095 |
|  |  | 2^nd^ generation PPI prescription duration | | N/A | 1.79 (0.59-5.39) | 0.303 |
| **Non asthma (n = 29,021) ‡** | | | |  |  |  |
|  |  | PPI prescription history | |  |  |  |
|  |  |  | Current PPI user | 176/561 (31.4) | 1.88 (1.55-2.28) | <0.001* |
|  |  |  | Past PPI user | 470/2,014 (23.3) | 1.33 (1.18-1.49) | <0.001* |
|  |  |  | PPI non-user (reference) | 4,372/26,446 (16.5) | 1 |  |
|  |  | PPI prescription duration | |  |  |  |
|  |  |  | ≥ 90 days | 109/360 (30.3) | 1.49 (1.15-1.93) | 0.002* |
|  |  |  | 30 – 89 days | 206/784 (26.3) | 1.51 (1.27-1.79) | <0.001* |
|  |  |  | < 30 days | 331/1,431 (23.1) | 1.38 (1.21-1.57) | <0.001* |
|  |  |  | PPI non-user (reference) | 4,372/26,446 (16.5) | 1 |  |
|  |  | 1^st^ generation PPI prescription duration | | N/A | 2.61 (1.57-4.31) | <0.001* |
|  |  | 2^nd^ generation PPI prescription duration | | N/A | 1.51 (0.76-3.01) | 0.240 |
| **Asthma (n = 6,949) ‡** | | | |  |  |  |
|  |  | PPI prescription history | |  |  |  |
|  |  |  | Current PPI user | 87/217 (40.1) | 1.39 (1.03-1.86) | 0.029* |
|  |  |  | Past PPI user | 243/694 (35.0) | 1.17 (0.98-1.40) | 0.089 |
|  |  |  | PPI non-user (reference) | 1,846/6,038 (30.6) | 1 |  |
|  |  | PPI prescription duration | |  |  |  |
|  |  |  | ≥ 90 days | 57/157 (36.3) | 1.00 (0.69-1.45) | 0.997 |
|  |  |  | 30 – 89 days | 100/257 (38.9) | 1.35 (1.03-1.78) | 0.029* |
|  |  |  | < 30 days | 173/497 (34.8) | 1.20 (0.99-1.47) | 0.070 |
|  |  |  | PPI non-user (reference) | 1,846/6,038 (30.6) | 1 |  |
|  |  | 1^st^ generation PPI prescription duration | | N/A | 2.05 (0.93-4.48) | 0.074 |
|  |  | 2^nd^ generation PPI prescription duration | | N/A | 0.45 (0.14-1.48) | 0.190 |
| **Non COPD (n = 33,627) ‡** | | | |  |  |  |
|  |  | PPI prescription history | |  |  |  |
|  |  |  | Current PPI user | 224/689 (32.5) | 1.71 (1.44-2.04) | <0.001* |
|  |  |  | Past PPI user | 630/2,465 (25.6) | 1.29 (1.17-1.43) | <0.001* |
|  |  |  | PPI non-user (reference) | 5,549/30,473 (18.2) | 1 |  |
|  |  | PPI prescription duration | |  |  |  |
|  |  |  | ≥ 90 days | 131/443 (29.6) | 1.21 (0.96-1.54) | 0.109 |
|  |  |  | 30 – 89 days | 274/938 (29.2) | 1.52 (1.31-1.78) | <0.001* |
|  |  |  | < 30 days | 449/1,773 (25.3) | 1.33 (1.19-1.49) | <0.001* |
|  |  |  | PPI non-user (reference) | 5,549/30,473 (18.2) | 1 |  |
|  |  | 1^st^ generation PPI prescription duration | | N/A | 2.27 (1.42-3.61) | 0.001* |
|  |  | 2^nd^ generation PPI prescription duration | | N/A | 1.13 (0.59-2.19) | 0.712 |
| **COPD (n = 2,343) ‡** | | | |  |  |  |
|  |  | PPI prescription history | |  |  |  |
|  |  |  | Current PPI user | 39/89 (43.8) | 1.67 (1.05-2.65) | 0.029* |
|  |  |  | Past PPI user | 83/243 (34.2) | 1.12 (0.82-1.51) | 0.484 |
|  |  |  | PPI non-user (reference) | 669/2,011 (33.3) | 1 |  |
|  |  | PPI prescription duration | |  |  |  |
|  |  |  | ≥ 90 days | 35/74 (47.3) | 1.92 (1.13-3.26) | 0.015* |
|  |  |  | 30 – 89 days | 32/103 (31.1) | 1.01 (0.64-1.59) | 0.963 |
|  |  |  | < 30 days | 55/155 (35.5) | 1.19 (0.84-1.70) | 0.330 |
|  |  |  | PPI non-user (reference) | 669/2,011 (33.3) | 1 |  |
|  |  | 1^st^ generation PPI prescription duration | | N/A | 3.56 (1.15-11.03) | 0.028* |
|  |  | 2^nd^ generation PPI prescription duration | | N/A | 1.35 (0.30-6.07) | 0.695 |
| **GERD non-treated (n = 29,931) ‡** | | | |  |  |  |
|  |  | PPI prescription history | |  |  |  |
|  |  |  | Current PPI user | 35/74 (47.3) | 1.64 (1.22-2.21) | 0.001* |
|  |  |  | Past PPI user | 32/103 (31.1) | 1.27 (1.08-1.48) | 0.003* |
|  |  |  | PPI non-user (reference) | 55/155 (35.5) | 1 |  |
|  |  | PPI prescription duration | |  |  |  |
|  |  |  | ≥ 90 days | 22/97 (22.7) | 1.02 (0.62-1.68) | 0.953 |
|  |  |  | 30 – 89 days | 70/301 (23.3) | 1.31 (0.99-1.72) | 0.058 |
|  |  |  | < 30 days | 200/794 (25.2) | 1.39 (1.17-1.64) | <0.001* |
|  |  |  | PPI non-user (reference) | 5,219/28,739 (18.2) | 1 |  |
|  |  | 1^st^ generation PPI prescription duration | | N/A | 1.68 (0.72-3.92) | 0.233 |
|  |  | 2^nd^ generation PPI prescription duration | | N/A | 1.08 (0.29-4.03) | 0.910 |
| **GERD Treated ≥ 1 time (n = 6,039) ‡** | | | |  |  |  |
|  |  | PPI prescription history | |  |  |  |
|  |  |  | Current PPI user | 197/553 (35.6) | 1.48 (1.21-1.81) | <0.001* |
|  |  |  | Past PPI user | 487/1,741 (28.0) | 1.04 (0.91-1.19) | 0.527 |
|  |  |  | PPI non-user (reference) | 999/3,745 (26.7) | 1 |  |
|  |  | PPI prescription duration | |  |  |  |
|  |  |  | ≥ 90 days | 144/420 (34.3) | 1.31 (1.03-1.66) | 0.028* |
|  |  |  | 30 – 89 days | 236/740 (31.9) | 1.27 (1.06-1.51) | 0.009* |
|  |  |  | < 30 days | 304/1,134 (26.8) | 1.02 (0.87-1.18) | 0.845 |
|  |  |  | PPI non-user (reference) | 999/3,745 (26.7) | 1 |  |
|  |  | 1^st^ generation PPI prescription duration | | N/A | 2.36 (1.43-3.89) | 0.001* |
|  |  | 2^nd^ generation PPI prescription duration | | N/A | 1.11 (0.56-2.19) | 0.761 |

Abbreviations: CCI, Charlson comorbidity index; CIs, confidence intervals; COPD, chronic obstructive pulmonary disease; CRS, chronic rhinosinusitis; DBP, diastolic blood pressure; GERD, gastro-esophageal reflux disease; NSAID, non-steroidal anti-inflammatory drug; ORs, odds ratios; PPI, proton pump inhibitor; SBP, systolic blood pressure

* Significance at *P* < 0.05

† Conditional logistic regression model was used. The model was stratified by age, sex, income, and residence region. The model was adjusted for total cholesterol, SBP, DBP, fasting blood glucose, obesity, smoking, alcohol consumption, CCI score, asthma, COPD, the number of GERD treatments, steroid/ NSAID/ H2 blocker prescription duration (model 3).

‡ Unconditional logistic regression model was used. The model was adjusted for age, sex, income, residence region, total cholesterol, SBP, DBP, fasting blood glucose, obesity, smoking, alcohol consumption, CCI score, asthma, COPD, the number of GERD treatments, steroid/ NSAID/ H2 blocker prescription duration.

**S5 Table** Subgroup analyses of PPI prescription history/PPI prescription duration/each generation PPI prescription duration for CRS according to covariates in model 3

| Characteristics | | | | No. of CRS/ No. of participants (%) | aORs (95% CIs) | P-value |
| --- | --- | --- | --- | --- | --- | --- |
| **Steroid prescription 0 days (n = 22,444) ‡** | | | |  |  |  |
|  |  | PPI prescription history | |  |  |  |
|  |  |  | Current PPI user | 100/363 (27.6) | 1.71 (1.34-2.20) | <0.001* |
|  |  |  | Past PPI user | 274/1,348 (20.3) | 1.19 (1.03-1.38) | 0.020* |
|  |  |  | PPI non-user (reference) | 3,207/20,733 (15.5) | 1 |  |
|  |  | PPI prescription duration | |  |  |  |
|  |  |  | ≥ 90 days | 64/235 (27.2) | 1.39 (1.00-1.92) | 0.049* |
|  |  |  | 30 – 89 days | 127/511 (24.9) | 1.56 (1.26-1.93) | <0.001* |
|  |  |  | < 30 days | 183/965 (19.0) | 1.15 (0.97-1.36) | 0.118 |
|  |  |  | PPI non-user (reference) | 3,207/20,733 (15.5) | 1 |  |
|  |  | 1^st^ generation PPI prescription duration | | N/A | 1.93 (0.99-3.77) | 0.054 |
|  |  | 2^nd^ generation PPI prescription duration | | N/A | 2.58 (1.01-6.58) | 0.047* |
| **Steroid prescription ≥ 1 days (n = 13,526) ‡** | | | |  |  |  |
|  |  | PPI prescription history | |  |  |  |
|  |  |  | Current PPI user | 163/415 (39.3) | 1.66 (1.33-2.05) | <0.001* |
|  |  |  | Past PPI user | 439/1,360 (32.3) | 1.28 (1.12-1.46) | <0.001* |
|  |  |  | PPI non-user (reference) | 3,011/11,751 (25.6) | 1 |  |
|  |  | PPI prescription duration | |  |  |  |
|  |  |  | ≥ 90 days | 102/282 (36.2) | 1.27 (0.96-1.68) | 0.097 |
|  |  |  | 30 – 89 days | 179/530 (33.8) | 1.34 (1.10-1.63) | 0.004* |
|  |  |  | < 30 days | 321/963 (33.3) | 1.38 (1.20-1.60) | <0.001* |
|  |  |  | PPI non-user (reference) | 3,011/11,751 (25.6) | 1 |  |
|  |  | 1^st^ generation PPI prescription duration | | N/A | 3.13 (1.78-5.50) | <0.001* |
|  |  | 2^nd^ generation PPI prescription duration | | N/A | 0.59 (0.27-1.31) | 0.195 |
| **NSAID prescription 0 days (n = 11,587) ‡** | | | |  |  |  |
|  |  | PPI prescription history | |  |  |  |
|  |  |  | Current PPI user | 18/111 (16.2) | 1.02 (0.59-1.75) | 0.954 |
|  |  |  | Past PPI user | 79/462 (17.1) | 1.26 (0.98-1.64) | 0.077 |
|  |  |  | PPI non-user (reference) | 1,453/11,014 (13.2) | 1 |  |
|  |  | PPI prescription duration | |  |  |  |
|  |  |  | ≥ 90 days | 14/85 (16.5) | 0.98 (0.52-1.85) | 0.944 |
|  |  |  | 30 – 89 days | 28/158 (17.7) | 1.29 (0.84-1.98) | 0.238 |
|  |  |  | < 30 days | 55/330 (16.7) | 1.24 (0.91-1.67) | 0.171 |
|  |  |  | PPI non-user (reference) | 1,453/11,014 (13.2) | 1 |  |
|  |  | 1^st^ generation PPI prescription duration | | N/A | 0.79 (0.18-3.45) | 0.758 |
|  |  | 2^nd^ generation PPI prescription duration | | N/A | 1.00 (0.17-5.93) | 0.998 |
| **NSAID prescription ≥ 1 days (n = 24,383) ‡** | | | | |  |  |
|  |  | PPI prescription history | |  |  |  |
|  |  |  | Current PPI user | 245/667 (36.7) | 1.74 (1.47-2.07) | <0.001* |
|  |  |  | Past PPI user | 634/2,246 (28.2) | 1.22 (1.10-1.36) | <0.001* |
|  |  |  | PPI non-user (reference) | 4,765/21,470 (22.2) | 1 |  |
|  |  | PPI prescription duration | |  |  |  |
|  |  |  | ≥ 90 days | 152/432 (35.2) | 1.37 (1.09-1.72) | 0.007* |
|  |  |  | 30 – 89 days | 278/883 (31.5) | 1.41 (1.21-1.65) | <0.001* |
|  |  |  | < 30 days | 449/1,598 (28.1) | 1.27 (1.13-1.43) | <0.001* |
|  |  |  | PPI non-user (reference) | 4,765/21,470 (22.2) | 1 |  |
|  |  | 1^st^ generation PPI prescription duration | | N/A | 2.78 (1.76-4.38) | <0.001* |
|  |  | 2^nd^ generation PPI prescription duration | | N/A | 1.09 (0.57-2.09) | 0.791 |
| **H2 blocker prescription 0 days (n = 18,460) ‡** | | | | |  |  |
|  |  | PPI prescription history | |  |  |  |
|  |  |  | Current PPI user | 50/174 (28.7) | 2.12 (1.49-3.03) | <0.001* |
|  |  |  | Past PPI user | 128/654 (19.6) | 1.28 (1.03-1.58) | 0.024* |
|  |  |  | PPI non-user (reference) | 2,658/17,632 (15.1) | 1 |  |
|  |  | PPI prescription duration | |  |  |  |
|  |  |  | ≥ 90 days | 29/106 (27.4) | 1.72 (1.04-2.84) | 0.033* |
|  |  |  | 30 – 89 days | 46/231 (19.9) | 1.28 (0.91-1.81) | 0.159 |
|  |  |  | < 30 days | 103/491 (21.0) | 1.45 (1.15-1.82) | 0.002* |
|  |  |  | PPI non-user (reference) | 2,658/17,632 (15.1) | 1 |  |
|  |  | 1^st^ generation PPI prescription duration | | N/A | 3.47 (1.37-8.80) | 0.009* |
|  |  | 2^nd^ generation PPI prescription duration | | N/A | 1.46 (0.47-4.51) | 0.510 |
| **H2 blocker prescription ≥ 1 days (n = 17,510) ‡** | | | | |  |  |
|  |  | PPI prescription history | |  |  |  |
|  |  |  | Current PPI user | 213/604 (35.3) | 1.49 (1.25-1.79) | <0.001* |
|  |  |  | Past PPI user | 585/2,054 (28.5) | 1.16 (1.04-1.29) | 0.010* |
|  |  |  | PPI non-user (reference) | 3,560/14,852 (24.0) | 1 |  |
|  |  | PPI prescription duration | |  |  |  |
|  |  |  | ≥ 90 days | 137/411 (33.3) | 1.20 (0.95-1.52) | 0.134 |
|  |  |  | 30 – 89 days | 260/810 (32.1) | 1.37 (1.16-1.61) | <0.001* |
|  |  |  | < 30 days | 401/1,437 (27.9) | 1.16 (1.02-1.31) | 0.020* |
|  |  |  | PPI non-user (reference) | 3,560/14,852 (24.0) | 1 |  |
|  |  | 1^st^ generation PPI prescription duration | | N/A | 1.97 (1.22-3.19) | 0.006* |
|  |  | 2^nd^ generation PPI prescription duration | | N/A | 1.04 (0.51-2.14) | 0.905 |

Abbreviations: CCI, Charlson comorbidity index; CIs, confidence intervals; COPD, chronic obstructive pulmonary disease; CRS, chronic rhinosinusitis; DBP, diastolic blood pressure; GERD, gastro-esophageal reflux disease; NSAID, non-steroidal anti-inflammatory drug; ORs, odds ratios; PPI, proton pump inhibitor; SBP, systolic blood pressure

* Significance at *P* < 0.05

† Conditional logistic regression model was used. The model was stratified by age, sex, income, and residence region. The model was adjusted for total cholesterol, SBP, DBP, fasting blood glucose, obesity, smoking, alcohol consumption, CCI score, asthma, COPD, the number of GERD treatments, steroid/ NSAID/ H2 blocker prescription duration (model 3).

‡ Unconditional logistic regression model was used. The model was adjusted for age, sex, income, residence region, total cholesterol, SBP, DBP, fasting blood glucose, obesity, smoking, alcohol consumption, CCI score, asthma, COPD, the number of GERD treatments, steroid/ NSAID/ H2 blocker prescription duration.
